# Prescription analgesia and adjuvant use by pain severity at admission among nursing home residents with non-malignant pain

**DOI:** 10.1007/s00228-020-02878-0

**Published:** 2020-05-03

**Authors:** Kate L. Lapane, Anne L. Hume, Reynolds A. Morrison, Bill M. Jesdale

**Affiliations:** 1 Department of Population and Quantitative Health Science, University of Massachusetts Medical School, 368 Plantation Street, Worcester, MA 01605 USA; 2 Department of Family Medicine, Alpert Medical School, Brown University, Memorial Hospital of Rhode Island, Providence, RI 02903 USA; 3 Department of Pharmacy Practice, College of Pharmacy, University of Rhode Island, Kingston, RI 02881 USA

**Keywords:** Nursing homes, Pain, Analgesics, Pain severity

## Abstract

**Objective:**

We estimated the use of prescribed analgesics and adjuvants among nursing home residents without cancer who reported pain at their admission assessment, in relation to resident-reported pain severity.

**Methods:**

Medicare Part D claims were used to define 3 classes of analgesics and 7 classes of potential adjuvants on the 21st day after nursing home admission (or the day of discharge for residents discharged before that date) among 180,780 residents with complete information admitted between January 1, 2011 and December 9, 2016, with no cancer diagnosis.

**Results:**

Of these residents, 27.9% reported mild pain, 46.6% moderate pain, and 25.6% reported severe pain. The prevalence of residents in pain without Part D claims for prescribed analgesic and/or adjuvant medications was 47.3% among those reporting mild pain, 35.7% among those with moderate pain, and 24.8% among those in severe pain. Among residents reporting severe pain, 33% of those ≥ 85 years of age and 35% of those moderately cognitively impaired received no prescription analgesics/adjuvants. Use of all classes of prescribed analgesics and adjuvants increased with resident-reported pain severity, and the concomitant use of medications from multiple classes was common.

**Conclusion:**

Among nursing home residents with recognized pain, opportunities to improve the pharmacologic management of pain, especially among older residents, and those living with cognitive impairments exist.

**Electronic supplementary material:**

The online version of this article (10.1007/s00228-020-02878-0) contains supplementary material, which is available to authorized users.

## Introduction

In the USA, there are ~ 1.7 million certified nursing home beds [1]. For the residents who live in this healthcare setting, pain is a common occurrence [2, 3]. If not treated appropriately, pain may be associated with complications such as depression, decreased social engagement, increased healthcare utilization and costs, increased functional limitations, and poor treatment outcomes [4–6]. The effective management of pain is key to improving or maintaining the quality of life of older adults.

In older adults, pharmacological treatment of pain can be challenging due to age-related physiologic changes, polypharmacy, and multimorbidity that may increase the risk of adverse events [7]. Cognitive and sensory impairments in old age also contribute to the inability of patients to effectively communicate about their pain with health professionals, which can negatively influence the types and intensity of treatments that are provided [8, 9]. Furthermore, uncertainty remains about the long-term safety and efficacy of common analgesics, and a lack of knowledge about both the cause of common pain syndromes [10] and the effectiveness of interventions to improve pain management [11].

Previously, we have shown that non-malignant pain recognition and management in nursing homes is sub-optimal, with up to one quarter of residents with daily pain not receiving any analgesics for treatment [12–14], despite clinical practice guidelines [15]. Using a national database of nursing home residents (2011–2016), this study aimed to provide a contemporary description of prescription analgesic and adjuvant use by pain severity among nursing home residents, to estimate the prevalence of lack of prescription analgesic use across levels of pain severity, and to identify factors associated with lack of prescription analgesics and/or adjuvants for residents with reported pain.

## Methods

This study was approved by the University of Massachusetts Medical School Institutional Review Board.

### Sample selection

We used the Minimum Data Set 3.0 [16, 17]. which is a valid and reliable tool completed by nursing home staff on virtually every nursing home resident in the USA. Used for research purposes [18], it includes a comprehensive admission assessment information of sociodemographics, active clinical diagnoses, and measures of functional [19] and cognitive status [20]. Supplemental Table 1 provides a detailed description of the sample selection.

### Pain medications

Although the effectiveness of non-pharmacological approaches to pain management is recognized [21], pharmacological approaches are the most commonly used to treat pain in older adults [4]. We focused on the use of analgesics or adjuvant medications for pain. We developed an expansive list of potential analgesics and adjuvants guided by trusted resources [15, 22] and reviewed by an expert in geriatric pharmacotherapy (AH). We categorized prescription analgesics as short-acting opioids (e.g., hydrocodone), long-acting opioids (e.g., fentanyl patches), and non-opioid analgesics (e.g., acetaminophen not in cold preparations or other combinations, celecoxib). We categorized prescription adjuvants into 7 categories: gabapentinoids, other anticonvulsant adjuvants (e.g., carbamazepine), SNRI antidepressants (e.g., duloxetine), tricyclic antidepressants (e.g., amitriptyline), muscle relaxants (e.g., clonazepam), systemic glucocorticoids (e.g., prednisone), and lidocaine patches. Residents with prescriptions with a day’s supply covering the index date were considered users of that medication. We then determined which classes of medications were used alone, or in combination with other classes of prescribed analgesics and/or adjuvants on the index date.

### Pain severity

The MDS 3.0 was changed significantly in October 2010 [23], with more opportunities for the “resident’s voice” to be heard [24]. Residents had documented pain in the lookback window of 5 days, and a self-assessment of pain severity, by one of two methods: a numeric pain intensity rating (J06a: “Please rate your worst pain over the last 5 days on a zero to ten scale, with zero being no pain and ten as the worst pain you can imagine.”), or a verbal descriptor scale (J06b: “Please rate the intensity of your worst pain over the last 5 days: mild; moderate; severe; very severe, horrible.”). Pain intensity ratings from 0 to 4 were tabulated with “mild” pain, pain intensity ratings from 5 to 7 were tabulated with “moderate” pain, and pain intensity ratings from 8 to 10 were tabulated with “severe” pain, as were residents who reported “very severe, horrible” pain. The MDS 3.0 provides the frequency of pain (i.e., rarely, occasionally, frequently, almost constantly), whether or not pain affects sleep, and whether or not pain limits day-to-day activities.

### Covariates

We selected demographics (age group, sex, race/ethnicity, admission source, and dependence in activities of daily living (ADL) [19]); potentially painful conditions from Section I (e.g., surgical wounds, arthritis, diabetes); conditions that may influence the communication of pain (e.g., cognitive function score [20], Alzheimer’s or other dementias); and conditions that may modify the pain experience (e.g., depression). Residents were classified as independent (ADL score 0–2), modified dependence (score 3–4), or dependent (score 5–6).

### Analytic strategy

With large sample sizes, trivial differences are often highly statistically significant. Instead, we considered absolute differences greater than 5% to be noteworthy. We described the distributions of key covariates and the use of monotherapy and combinations of analgesics or adjuvants by level of pain severity. The prevalence of a lack of prescribed analgesic/adjuvant medications was estimated, stratified by pain severity. We used a linear modeling approach (logarithmic link function with a Poisson distribution [25]) to estimate adjusted prevalence ratios with 95% confidence limits, overall and stratified by resident-reported pain severity.

## Results

**Fig. 1:**
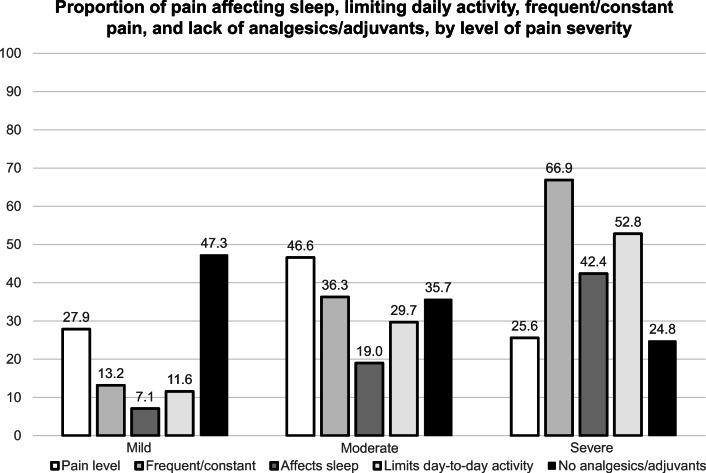
Proportion of pain affecting sleep, limiting daily activity, frequent/constant pain, and lack of analgesics/adjuvants, by level o pain severity

Of 180,780 nursing home residents with documented pain (Fig. 1), 27.9% reported mild pain, 46.6% moderate pain, and 25.6% severe pain. Among the 27.9% reporting mild pain, 47.3% had no Part D claims for analgesics or adjuvants; 22.7% reported their pain frequency as rarely, 63.4% as occasionally, 10.7% as frequently, and 2.5% as almost constantly. Among the 46.6% reporting moderate pain, 35.7% had no Part D claims for analgesic or adjuvants; 7.2% reported their pain frequency as rarely, 55.9% as occasionally, 30.1% as frequently, and 6.2% as almost constantly. Among the 25.6% reporting severe pain, 3.5% reported their pain frequency as rarely, 28.7% as occasionally, 44.0% as frequently, and 22.9% as almost constantly, with 42.4% reporting that pain affects sleep and 52.8% indicating that pain limits day-to-day activities. Among those reporting severe pain, 24.8% had no Part D claims for analgesic or adjuvants.

While distributions of sex, race/ethnicity, activities of daily living, and potentially painful conditions were similar across level of pain severity, the distribution of age varied across levels of pain severity with 44.6% of those in mild pain ≥ 85 years of age whereas 34.0% of those in severe pain were ≥ 85 years of age (Table 1). The distribution of cognitive impairment and active diagnoses of Alzheimer’s or other dementias were different across levels of pain severity.

**Table 1:** Characteristics of newly admitted nursing home residents by resident-reported pain severity

	Mild pain (*n* = 50,440)	Moderate pain (*n* = 84,195)	Severe pain (*n* = 46,145)
	Percentage		
Age group (years)			
50 to 64	8.0	13.3	14.3
65 to 74	17.4	19.2	23.0
75 to 84	30.0	30.0	28.7
≥ 85	44.6	40.5	34.0
Men	26.4	24.9	23.8
Race/ethnicity*			
Hispanic of any race(s)	4.6	4.7	4.0
Non-Hispanic, White	84.3	84.4	86.4
Non-Hispanic, Black	7.9	7.7	7.3
Admission source			
Acute care hospital	42.1	48.6	51.9
Another nursing home or swing bed	14.5	13.2	12.1
Community	41.4	36.4	34.2
Another source	1.9	1.8	1.8
Activities of daily living			
Independent	29.3	26.2	25.0
Modified dependence	56.4	57.4	57.1
Dependent	14.3	16.4	17.9
Potentially painful conditions			
Heart failure	18.1	18.6	19.4
Respiratory failure	1.1	1.2	1.4
Surgical wounds or wound infections	6.5	9.0	9.7
Arthritis	35.5	38.0	39.7
Osteoporosis	17.0	18.1	18.8
Recent fracture	12.1	17.2	19.1
Mouth or face pain	1.5	1.7	2.5
Gastroesophageal reflux disorder	35.6	37.3	39.6
Ulcerative colitis, Crohn’s or irritable bowel	0.9	1.0	1.2
Swallowing disorder	3.3	3.2	3.5
High (2+) grade pressure ulcers	7.0	8.3	9.2
Foot problems	1.9	2.2	2.5
Other open lesions, or burns	1.7	1.8	2.0
Diabetes	31.5	33.0	35.2
Conditions that may influence the expression or recognition of pain			
Cognitive Function Score			
Cognitively intact	47.6	55.3	62.2
Mildly impaired	27.9	26.6	25.1
Moderately/severely impaired	24.4	18.0	12.8
Alzheimer’s or other dementia	35.3	27.6	21.7
Use of antipsychotics or hypnotics in past 7 days	21.4	21.4	23.1
Conditions that may modify the experience of pain			
Depression	40.0	41.6	44.5
Anxiety disorder	24.5	26.1	29.2

*Few residents reported being non-Hispanic, American Indian/Alaska Native (0.8% of each pain category), non-Hispanic Asian (2.1% of those in mild pain, 2.1% of those in moderate pain, and 1.1% of those in severe pain), non-Hispanic Pacifica Islander (0.3% of each pain category), and multiracial (0% of those in mild pain, 0.1% of those in moderate pain, 0.1% of those in severe pain)

Short-acting opioids were commonly used (Table 2: 23.3% among those with mild pain, 34.9% of those with moderate pain, and 46.5% of those with severe pain). Regardless of the level of pain severity, more than half receiving short-acting opioids used them in combination with long-acting opioids, non-opioid analgesics, and/or potential adjuvants. For example, among short-acting opioid users in severe pain, 54% also used potential adjuvants (25.2% of the whole sample), 20% also used long-acting opioids (9.3% of the whole sample), and 14% used non-opioid analgesics (6.6% of the whole sample). Non-opioid analgesics were used in 10.6% of those with mild pain, 12.0% of those in moderate pain, and 13.2% of those in severe pain. Potential adjuvant medications were used by 34.0% of those with mild pain and 48.0% of those with severe pain, with gabapentinoids most common. Use of other anticonvulsants was < 5%, regardless of level of pain severity. SNRI antidepressants were used in 7.8% of residents with mild pain, 9.6% of residents with moderate pain, and 12.3% of residents with severe pain. Muscle relaxants were used in 5.4% of residents with mild pain, 6.4% in those with moderate pain, and 7.8% of those reporting severe pain. No pain management strategies were documented for 19.8% of those in mild pain, 12.0% of those in moderate pain, and 11.4% of those in severe pain (Supplemental Table 2).

**Table 2:** Use of analgesics and/or potential adjuvants 21 days after admission (or discharge date*), by resident-reported pain severity

	Mild pain (*n* = 50,440)	Moderate pain (*n* = 84,195)	Severe pain (*n* = 46,145)
	Percentage		
Prescription analgesics	33.4	46.4	59.2
Short-acting opioids	23.3	34.9	46.5
As monotherapy	10.5	14.1	15.8
In combination with long-acting opioids	2.1	4.6	9.3
In combination with non-opioid analgesics	3.1	4.9	6.6
In combination with potential adjuvants	10.6	17.1	25.2
Long-acting opioids	5.1	9.6	16.5
As monotherapy	1.3	1.9	2.6
In combination with non-opioid analgesics	0.6	1.3	2.3
In combination with potential adjuvants	2.9	5.8	10.3
Non-opioid analgesics	10.6	12.0	13.2
As monotherapy	4.5	3.9	3.1
In combination with potential adjuvants	4.4	5.6	7.2
Potential adjuvant prescription medications	34.0	40.5	48.0
Gabapentinoids	16.3	20.6	25.9
As monotherapy	5.7	5.5	5.0
In combination with prescription analgesics	7.7	12.2	17.8
Other anticonvulsant adjuvants	4.4	4.4	5.0
As monotherapy	1.8	1.3	1.1
In combination with prescription analgesics	1.5	2.2	3.0
SNRI antidepressants	7.8	9.6	12.3
As monotherapy	2.3	2.0	1.6
In combination with prescription analgesics	3.8	5.8	8.8
Tricyclic antidepressants	2.5	3.0	3.8
As monotherapy	0.8	0.7	0.5
In combination with prescription analgesics	1.1	1.7	2.6
Systemic glucocorticoids	4.6	5.8	7.1
As monotherapy	1.8	1.7	1.3
In combination with prescription analgesics	2.6	3.9	5.6
Muscle relaxants	5.4	6.4	7.8
As monotherapy	1.5	1.2	1.0
In combination with prescription analgesics	2.0	3.2	4.9
Lidocaine patches	3.7	5.4	6.9
As monotherapy	1.2	1.3	1.2
In combination with prescription analgesics	1.8	3.3	4.9

*The index date was either the 21st day after admission or the date of discharge for the ~ 15% of residents discharged from the nursing home in the first 21 days. We selected 21 days because the mode of MDS assessment completion was 7 days after admission, giving nursing home staff ample opportunity to respond to resident-reported pain

Table 3 shows that increasing pain severity was inversely associated with lack of receipt of pharmacological pain medications (adjusted PR moderate versus mild, 0.80 (95% CI 0.79–0.82), adjusted PR severe versus mild, 0.60 (95% CI 0.59–0.62)). Factors placing residents at greater risk of not receiving prescription medications included advanced age (adjusted PR age ≥ 85 years versus 50–64 years, 2.00 (95% CI 1.93–2.08)), moderate to severe cognitive impairment (adjusted PR, 1.20 (95% CI 1.17–1.22)), and having Alzheimer’s disease or other dementias (adjusted PR, 1.14 (95% CI 1.12–1.17)). Estimates of prevalence ratios were similar across the three levels of pain severity (Supplemental Table 3).

**Table 3:** Association between resident characteristics and lack of prescription analgesics/adjuvant medications among residents reporting pain

	Prevalence of no prescription analgesics or adjuvant medications, by resident-reported pain severity			Among all residents reporting pain
	Mild pain (*n* = 50,440)	Moderate pain (*n* = 84,195)	Severe pain (*n* = 46,145)	Adjusted** prevalence ratio (95% confidence interval)
	Percentage			
Resident-reported pain severity				
Mild	100			1.0
Moderate		100		0.80 (0.79–0.82)
Severe			100	0.60 (0.59–0.62)
Age group (years)				
50 to 64	28.0	19.8	12.7	1.0
65 to 74	37.2	26.8	19.0	1.35 (1.30–1.40)
75 to 84	46.9	36.0	25.6	1.71 (1.65–1.78)
85 and older	55.1	43.7	33.2	2.00 (1.93–2.08)
Men	48.9	37.3	26.9	1.12 (1.10–1.14)
Race/ethnicity				
Hispanic of any race(s)	47.2	37.3	27.7	1.03 (1.00–1.07)
Non-Hispanic, White	47.5	35.7	24.5	1.0
Non-Hispanic, Black	43.3	33.2	26.1	1.02 (0.99–1.05)
American Indian/Alaska Native alone	50.8	35.7	24.1	1.06 (0.97–1.15)
Asian alone	52.1	41.4	35.7	1.05 (0.99–1.11)
Pacific Islander alone	57.1	43.3	31.0	1.13 (1.00–1.27)
Multiracial	29.2	24.5	21.1	0.78 (0.54–1.14)
Admission source				
Acute care hospital	50.1	38.1	26.6	1.03 (1.02–1.05)
Another nursing home or swing bed	40.9	29.5	21.3	0.93 (0.90–0.95)
Community	46.8	35.6	24.4	1.0
Another source	45.5	35.9	27.5	1.19 (1.12–1.26)
Activities of daily living				
Independent	48.0	35.6	23.8	1.0
Modified dependence	47.8	36.3	25.4	0.97 (0.96–0.99)
Dependent	44.3	33.6	24.4	0.93 (0.90–0.95)
Potentially painful conditions				
Heart failure	45.8	34.8	25.1	1.00 (0.98–1.02)
Respiratory failure	33.2	25.9	15.3	0.88 (0.81–0.96)
Surgical wounds or wound infections	43.6	33.9	25.0	1.04 (1.01–1.08)
Arthritis	42.5	31.7	22.0	0.82 (0.81–0.83)
Osteoporosis	46.6	34.3	23.1	0.93 (0.91–0.95)
Recent fracture	48.0	38.3	27.4	1.01 (0.99–1.04)
Mouth or face pain	47.6	33.4	25.9	1.01 (0.95–1.07)
Gastroesophageal reflux disorder	41.6	31.1	21.5	0.89 (0.88–0.91)
Ulcerative colitis, Crohn’s or irritable bowel	41.1	31.8	21.2	0.96 (0.89–1.04)
Swallowing disorder	48.5	38.3	27.5	1.04 (1.00–1.09)
High (2+) grade pressure ulcers	41.5	31.6	22.2	0.90 (0.88–0.93)
Foot problems	43.4	29.9	22.2	0.94 (0.88–0.99)
Other open lesions, or burns	47.0	34.1	20.7	0.97 (0.92–1.03)
Diabetes	43.0	32.4	22.7	0.96 (0.95–0.98)
Cognitive Function Score				
Cognitively intact	42.3	31.4	21.7	1.0
Mildly impaired	48.8	38.1	27.6	1.10 (1.08–1.12)
Moderately or severely impaired	55.4	45.2	34.9	1.20 (1.17–1.22)
Alzheimer’s or other dementia	52.7	42.1	31.0	1.14 (1.12–1.17)
Use of antipsychotics or hypnotics in past 7 days	43.1	30.6	20.3	0.94 (0.92–0.96)
Conditions that may modify the experience of pain				
Depression	39.2	28.1	18.8	0.78 (0.77–0.80)
Anxiety disorder	39.0	28.1	17.8	0.85 (0.83–0.86)

*Adjusted for all resident characteristics shown on the table

## Discussion

This study attempted to shed more light on the prescription pain management strategies used in nursing home residents with documented pain. This study did *not* include residents without pain documented because they either did not experience pain in the 5 days preceding the MDS assessment or the pain management strategies used adequately controlled their pain. We previously have noted that advanced age, race/ethnicity, cognitive impairment, and dementia were inversely associated with persistent and intermittent pain [3, 12, 13], findings aligned with research by others [26, 27]. We demonstrated that among those who self-reported pain, those reporting severe pain were less likely to be of advanced age or to have cognitive impairment relative to those reporting mild pain. Exploring whether stoicism in advanced age explains our findings, as has been reported by others [28], is beyond the scope of our data. Concerns about MDS 3.0 pain measures have been noted [29, 30]. The MDS 3.0 offered significant improvements to capturing the resident experience [23] and the vast majority of residents provide self-reported information [24]. Continued efforts to improve the recognition of pain in nursing home residents is warranted.

Among nursing home residents with recognized pain, one in four rated their pain as severe, with two-thirds of residents with severe pain noting it occurred frequently or almost constantly. Pain impacted residents’ sleep and limited residents’ ability to do day-to-day activities, and this increased with level of pain severity. Prescribed analgesics and adjuvants increased markedly with pain severity for all groups of analgesics and adjuvants considered. However, 24.8% lacked prescription medications for pain among those with identified severe pain. Residents aged ≥ 85 years were least likely to receive prescribed analgesics or adjuvants as compared with younger nursing home residents. Cognitively impaired residents were more likely to endure their pain without the use of prescribed analgesics or adjuvants relative to residents with mild cognitive impairment.

We found that in those with pain recognized and documented by nursing home staff, lack of prescription analgesics was common, although less frequent with increased severity of pain. In the USA, nursing homes are required by law (42 CFR §483.60) to provide pharmaceutical services to meet the needs of each resident [31]. Our findings documenting the extent to which residents with documented pain had no Part D claims for prescription analgesics, indicate that opportunities to enhance compliance with this federal legislation are plentiful. Advanced age and level of cognitive impairment were associated with decreased use of prescription pain medications, consistent with what others have shown [32, 33]. The US Centers for Medicare & Medicaid Services provided revised guidance for meeting compliance in the evaluation and management of pain in nursing home residents (i.e., F-Tag 309) in 2009. Such administrative initiatives appear to fall short. The extent to which these findings have been influenced by federal efforts to address opioid abuse is unknown [34]. Despite these efforts, improving pain management in nursing home residents deserving of relief from suffering and dignity in care [35] is imperative.

Consistent with previous research, we found that opioid use was common [36], as was use of gabapentinoids [37, 38]. With increased severity of pain, we see increased use of combination therapies, suggesting that nursing homes often aggressively attempt to manage pain. Our data do not permit us to evaluate the extent to which the “right” pain medications or combination of analgesics and/or adjuvants are given to the “right” residents at the “right” time [39]. The Institute of Medicine’s report “Relieving Pain in America” challenged researchers to implement a cultural transformation to better understand pain and its management [40]. Foundational knowledge about how best to support nursing homes to continue improving pain management among those in life’s final chapter is needed.

Our data should be interpreted with caution as we were unable to include those receiving Medicare managed care or post-acute rehabilitation. Extrapolation of the findings from this study to residents covered by managed care, or to residents receiving post-acute rehabilitation services, should be undertaken with caution. Residents without Part D claims for analgesics or adjuvants may have received non-prescription pain relief and/or non-pharmacologic pain management. The MDS 3.0 lacks specific details about what non-pharmacological approaches or over the counter medications were used for pain. The use of prescribed adjuvant medications (e.g., SNRI antidepressants, systemic glucocorticoids, anticonvulsants) may have been for indications other than pain.

## Conclusions

Many nursing home residents with pain receive no prescription pharmacological management 3 weeks into their stay. Those with cognitive impairment and those with advanced age (≥ 85 years) had the greatest likelihood of no treatment. In those whose pain is treated, use of combination analgesics +/- adjuvants increased with severity of pain. Understanding whether lack of prescription pharmacological management of pain among those with moderate to severe pain at admission reflects resident preference, clinician uncertainty given the lack of a strong evidence base regarding risks and benefits in nursing home residents, or other modifiable factors is warranted to provide “personalized” pain management to a population deserving of improved quality of life [41].

## Electronic supplementary material

ESM 1 (DOCX 16 kb)
