# Supplementary material for: Prescription analgesia and adjuvant use by pain severity at admission among nursing home residents with non-malignant pain
Source: Eur J Clin Pharmacol. 2020 May 3;76(7):1021–8. doi: 10.1007/s00228-020-02878-0 (PMC7306024; doi:10.1007/s00228-020-02878-0)
Supplement: Supplementary file 1 — (DOCX 16 kb) [file 228_2020_2878_MOESM1_ESM.docx]

**Supplemental Table 1. Study sample flowchart**

|  | Description | Rationale | Sample size |
| --- | --- | --- | --- |
| Inclusion | Nursing home resident admissions between 1/1/2011 and 12/9/2016 were potentially eligible if there was no evidence of a skilled nursing facility (SNF) stay within 90 days before admission, or 30 days after admission | We excluded SNF stays because medications are bundled into the per diem rate. As such, Part D claims are not filed for prescription medications administered during the SNF stay. | n=3,311,902 admissions |
| Exclusions | <50 years old  residents with cancer, those who were comatose, and those admitted for developmental disabilities or mental retardation. | We focused on medication use in older adults. | n=2,351,236 admissions |
| Exclusions | resident-reported pain presence and pain severity | Key stratification variable3 | n=1,149,537 admissions |
| Exclusions | Missing covariate data |  | n=1,140,688 admissions |
| Exclusion | Not enrolled in Part A/B/D Medicare Fee for Service during the month of admission | We used these data to define medication use. | n=214,543 admissions,  from which we randomly selected one assessment  n=195, 399 resident |
| Exclusion | No evidence of any Part D claim in the first 21 days after admission | We selected 21 days as the index date (or discharge date if discharged before 21 days) as we believed this gave nursing home staff ample opportunity to respond to MDS 3.0 documented pain. | n=180,780 |

**Supplemental Table 2. MDS variables of pain management strategies by pain severity level**

|  | Mild  Pain  (n=50,440) | Moderate Pain  (n=84,195) | Severe Pain  (n=46,145) |
| --- | --- | --- | --- |
|  | *Percentage* | | |
| Scheduled Prescription analgesics | 21.8 | 25.0 | 28.1 |
| Pro re nata pain medication regimen | 66.3 | 77.5 | 80.0 |
| Non-pharmacological intervention | 31.0 | 37.8 | 40.3 |
| None of the above | 19.8 | 12.0 | 11.4 |

**Supplemental Table 3. Mutually adjusted prevalence ratios of no prescription analgesia or adjuvant medications, stratified by resident-reported pain severity.**

|  | Mild Pain | Moderate Pain | Severe Pain |
| --- | --- | --- | --- |
|  | Prevalence Ratio (95% Confidence interval) | Prevalence Ratio (95% Confidence interval) | Prevalence Ratio (95% Confidence interval) |
|  | % | % | % |
| Age group |  |  |  |
| 50 to 64 | (ref) | (ref) | (ref) |
| 65 to 74 | 1.31 (1.22 – 1.40) | 1.31 (1.24 – 1.39) | 1.45 (1.34 – 1.57) |
| 75 to 84 | 1.59 (1.49 – 1.70) | 1.69 (1.61 – 1.79) | 1.90 (1.75 – 2.05) |
| 85 and older | 1.82 (1.70 – 1.94) | 1.97 (1.87 – 2.08) | 2.36 (2.18 – 2.55) |
| Male | 1.10 (1.06 – 1.13) | 1.12 (1.09 – 1.15) | 1.16 (1.11 – 1.22) |
| Race / ethnicity |  |  |  |
| Hispanic of any race(s) | 1.00 (0.94 – 1.06) | 1.04 (0.99 – 1.10) | 1.10 (1.01 – 1.21) |
| Not Hispanic |  |  |  |
| White alone | (ref) | (ref) | (ref) |
| Black alone | 0.97 (0.92 – 1.02) | 1.01 (0.97 – 1.06) | 1.17 (1.09 – 1.25) |
| American Indian / Alaska Native alone | 1.08 (0.94 – 1.24) | 1.03 (0.91 – 1.17) | 1.10 (0.89 – 1.35) |
| Asian alone | 1.02 (0.93 – 1.11) | 1.03 (0.96 – 1.12) | 1.24 (1.07 – 1.43) |
| Pacific Islander alone | 1.16 (0.95 – 1.42) | 1.13 (0.95 – 1.35) | 1.02 (0.73 – 1.41) |
| Multiracial | 0.62 (0.30 – 1.30) | 0.79 (0.45 – 1.39) | 1.03 (0.52 – 2.07) |
| Admission source |  |  |  |
| Acute care hospital | 1.02 (0.99 – 1.05) | 1.03 (1.00 – 1.06) | 1.07 (1.03 – 1.12) |
| Another nursing home or swing bed | 0.92 (0.88 – 0.96) | 0.91 (0.87 – 0.94) | 0.99 (0.93 – 1.06) |
| Community | (ref) | (ref) | (ref) |
| Another source | 1.08 (0.98 – 1.19) | 1.20 (1.10 – 1.31) | 1.37 (1.20 – 1.57) |
| Activities of daily living |  |  |  |
| 0-2 Independent | (ref) | (ref) | (ref) |
| 3-4 Modified dependence | 0.97 (0.94 – 1.00) | 0.97 (0.95 – 1.00) | 0.98 (0.94 – 1.03) |
| 5-6 Dependent | 0.93 (0.89 – 0.97) | 0.92 (0.88 – 0.95) | 0.95 (0.90 – 1.01) |
| Potentially painful conditions |  |  |  |
| Heart failure | 0.98 (0.95 – 1.02) | 1.00 (0.97 – 1.03) | 1.03 (0.98 – 1.08) |
| Respiratory failure | 0.87 (0.75 – 1.01) | 0.92 (0.81 – 1.04) | 0.82 (0.67 – 1.00) |
| Surgical wounds or wound infections | 1.00 (0.94 – 1.05) | 1.04 (0.99 – 1.08) | 1.13 (1.06 – 1.21) |
| Arthritis | 0.83 (0.81 – 0.86) | 0.82 (0.80 – 0.84) | 0.80 (0.77 – 0.83) |
| Osteoporosis | 0.97 (0.93 – 1.00) | 0.92 (0.89 – 0.95) | 0.86 (0.82 – 0.91) |
| Recent fracture | 0.98 (0.94 – 1.02) | 1.02 (0.99 – 1.05) | 1.05 (1.00 – 1.10) |
| Mouth or face pain | 1.01 (0.91 – 1.12) | 0.97 (0.89 – 1.06) | 1.09 (0.97 – 1.22) |
| Gastroesophageal reflux disorder | 0.89 (0.86 – 0.91) | 0.89 (0.87 – 0.91) | 0.90 (0.87 – 0.94) |
| Ulcerative colitis, Crohn’s or irritable bowel | 0.93 (0.81 – 1.07) | 0.99 (0.88 – 1.11) | 0.96 (0.80 – 1.15) |
| Swallowing disorder | 1.02 (0.95 – 1.09) | 1.05 (0.98 – 1.12) | 1.08 (0.98 – 1.18) |
| High (2+) grade pressure ulcers | 0.90 (0.85 – 0.95) | 0.91 (0.87 – 0.95) | 0.89 (0.83 – 0.96) |
| Foot problems | 0.98 (0.89 – 1.08) | 0.90 (0.82 – 0.98) | 0.95 (0.84 – 1.07) |
| Other open lesions, or burns | 1.01 (0.92 – 1.12) | 0.97 (0.89 – 1.06) | 0.90 (0.78 – 1.04) |
| Diabetes | 0.95 (0.93 – 0.98) | 0.96 (0.94 – 0.99) | 0.97 (0.94 – 1.02) |
| Conditions that may influence the expression or recognition of pain | | |  |
| Cognitive Function Score |  |  |  |
| Cognitively intact | (ref) | (ref) | (ref) |
| Mildly impaired | 1.07 (1.04 – 1.10) | 1.10 (1.07 – 1.13) | 1.13 (1.08 – 1.18) |
| Moderately or severely impaired | 1.16 (1.12 – 1.20) | 1.21 (1.17 – 1.25) | 1.27 (1.20 – 1.34) |
| Alzheimer’s or other dementia | 1.10 (1.07 – 1.14) | 1.16 (1.13 – 1.20) | 1.19 (1.14 – 1.25) |
| Use of antipsychotics or hypnotics in past 7 days | 0.97 (0.94 – 1.00) | 0.93 (0.90 – 0.96) | 0.92 (0.87 – 0.96) |
| Conditions that may modify the experience of pain |  |  |  |
| Depression | 0.82 (0.79 – 0.84) | 0.77 (0.75 – 0.79) | 0.75 (0.72 – 0.79) |
| Anxiety disorder | 0.87 (0.84 – 0.90) | 0.86 (0.83 – 0.88) | 0.79 (0.76 – 0.83) |
